# Supplementary material for: Lung and blood early biomarkers for host-directed tuberculosis therapies: Secondary outcome measures from a randomized controlled trial
Source: PLoS One. 2022 Feb 4;17(2):e0252097. doi: 10.1371/journal.pone.0252097 (PMC8815935; doi:10.1371/journal.pone.0252097)
Supplement: Attachment — Submitted filename: Rebuttal.docx [file pone.0252097.s001.docx]

27 April 2021

Re: PONE-D-21-03831

Thank you very much for the opportunity to revise this manuscript. We have addressed the questions as follows:

Editor’s comments:

*If applicable, we recommend that you deposit your laboratory protocols in protocols.io to enhance the reproducibility of your results.*

We have placed the protocol online and have included a link to its URL in the text.

Please review your reference list to ensure that it is complete and correct. If you have cited papers that have been retracted, please include the rationale for doing so in the manuscript text, or remove these references and replace them with relevant current references. Any changes to the reference list should be mentioned in the rebuttal letter that accompanies your revised manuscript. If you need to cite a retracted article, indicate the article’s retracted status in the References list and also include a citation and full reference for the retraction notice.

*1. Please ensure that your manuscript meets PLOS ONE's style requirements, including those for file naming. The PLOS ONE style templates can be found at*

https://journals.plos.org/plosone/s/file?id=wjVg/PLOSOne_formatting_sample_main_body.pdf and

https://journals.plos.org/plosone/s/file?id=ba62/PLOSOne_formatting_sample_title_authors_affiliations.pdf

*2. In your Data Availability statement, you have not specified where the minimal data set underlying the results described in your manuscript can be found.*

We are currently working with the Critical Path Institute to place the full study dataset online as a part of the ERA4TB Consortium. We would like to include the full PET/CT images. This has proven challenging, as the scans are about 1TB in total. The text has been modified to indicate this.

Reviewers’ comments:

*Reviewer #1: For Tables 2 - 5, please include the statistical analysis method used to calculate the CI's and p-values in either the description of the table or in the table footnote.*

This has been added.

*The text in the statistical analysis section mentions that ANCOVA was used and that analyses were adjusted for the relevant baseline characteristics. Please indicate what these characteristics are. It is not appropriate to leave it up to the reader to guess them.*

This has been added.

*Reviewer #2: The persistance of tuberculosis damage on the lung has a great impact on patients' quality of life and prognosis. The final aim of the work was to identify host directed drugs that would be able to improve recovery of lung function. The authors published a first paper using FEV1 as a surrogate of HTD efficacy. It worked, but the impact wasn't demonstrable before the end of the treatment. The aim of the second paper was to select biomarkers that would reflect early improvement due to the tested drugs. Several positive results are reported. However, it seems difficult to evaluate in the same experiment the drugs and the biomarkers. If the drugs are not working well, how could the authors identify the best biomarker? This point could be discussed in the discussion.*

It is generally thought that the most valuable biomarkers are agnostic as to the mechanism of the intervention. We did not identify any biomarkers meeting this standard. The text has been revised to reflect this.

*Some minor points could be modified:*

*the results about FEV1 are already published and reported in the introduction. Why reporting them in the results part of the abstract?*

We have edited the abstract to keep this to a minimum.

*Auranofin has a direct microbicidal activity as the authors reported. Could this property explain the enhancement of mycobacteria killing?*

Yes, we believe this is the case. The text has been revised to clarify this point.

*The trend of its effect on CRP is , as the authors say, not significant. Has it to be considered?*

Yes, we have edited the text to emphasize the effect did not reach statistical significance.
